# Overexpression of *Shox2* Leads to Congenital Dysplasia of the Temporomandibular Joint in Mice

**DOI:** 10.3390/ijms150813135

**Published:** 2014-07-24

**Authors:** Xihai Li, Wenna Liang, Hongzhi Ye, Xiaping Weng, Fayuan Liu, Xianxiang Liu

**Affiliations:** 1 Academy of Integrative Medicine, Fujian University of Traditional Chinese Medicine, Fuzhou 350122, China; E-Mails: lixihaifz@gmail.com (X.L.); yehzfjzy@gmail.com (H.Y.); wengxiapingfz@gmail.com (X.W.); liufayuanfz@gmail.com (F.L.); 2 Research Base of Traditional Chinese Medicine Syndrome, Fujian University of Traditional Chinese Medicine, Fuzhou 350122, China; E-Mail: liangwennafz@gmail.com

**Keywords:** *Shox2*, temporomandibular joint, articular cartilage, extracellular matrix, matrix metalloproteinase

## Abstract

Our previous study reported that inactivation of *Shox2* led to dysplasia and ankylosis of the temporomandibular joint (TMJ), and that replacing *Shox2* with human *Shox* partially rescued the phenotype with a prematurely worn out articular disc. However, the mechanisms of *Shox2* activity in TMJ development remain to be elucidated. In this study, we investigated the molecular and cellular basis for the congenital dysplasia of TMJ in *Wnt1-Cre*; *pMes-stop Shox2* mice. We found that condyle and glenoid fossa dysplasia occurs primarily in the second week after the birth. The dysplastic TMJ of *Wnt1-Cre*; *pMes-stop Shox2* mice exhibits a loss of Collagen type I, Collagen type II, Ihh and Gli2. *In situ* zymography and immunohistochemistry further demonstrate an up-regulation of matrix metalloproteinases (MMPs), MMP9 and MMP13, accompanied by a significantly increased cell apoptosis. In addition, the cell proliferation and expressions of Sox9, Runx2 and Ihh are no different in the embryonic TMJ between the wild type and mutant mice. Our results show that overexpression of *Shox2* leads to the loss of extracellular matrix and the increase of cell apoptosis in TMJ dysplasia by up-regulating MMPs and down-regulating the Ihh signaling pathway.

## 1. Introduction

Temporomandibular joint (TMJ), a mammalian synovial joint essential for jaw function, consists of the fibrocartilaginous disc and the condyle, derived from a secondary cartilage by endochondral ossification, and the glenoid fossa arising from the otic capsule through intramembranous ossification [1,2]. During the processes of TMJ development, a large number of transcription factors and growth factors have been implicated in the development of primary cartilage and endochondral ossification, such as *Sox9*, *Runx2*, and *Ihh* [3,4,5]. The Hedgehog (Hh) signaling pathway plays a pivotal role in digit joint formation, which has been implicated in the initial formation and separation of the articular disc from the apex of condyle, and the proper structure and tissue homeostasis of the TMJ, as evidenced by the absence of the articular disc formation in *Ihh*^−/−^ and *Gli2*^−/−^ mutant mice, and the partial disc ankylosis and TMJ dysplasia in ablation of *Ihh* in the cartilage of neonatal mice [3,6,7].

*Short stature homeobox 2* (*Shox2*) is expressed in the condylar chondrocytes and glenoid fossa of the developing TMJ, and there is an absence of *Shox2* expression in the hypertrophic chondrocyte zone. Previous reports showed that targeted inactivation of *Shox2* leads to severe defects in a number of developing organs including heart, palate, and limb, and that TMJ exhibits dysplasia and ankylosis in mice [8,9,10,11,12]. We also reported that although *Shox2*^Shox–KI/KI^ mice did not exhibit TMJ dysplasia and ankylosis at birth, a phenotype observed in mice carrying inactivation of *Shox2* in the cranial neural crest lineage, the mice did develop a different TMJ defect, a premature wearing out of the articular disc postnatally [2,13,14]. These results support a role proposed for the *Shox2* genes in skeletogenesis and TMJ. However, the precise mechanisms of *Shox2* on the development and function of the TMJ at the molecular and cellular level remain poorly understood. In this study, we sought to investigate the effect of overexpression of *Shox2* on TMJ development to explore the mechanism of the TMJ dysplasia in the *Wnt1-Cre*; *pMes-stop Shox2* mice.

## 2. Results

### 2.1. Wnt1-Cre; pMes-stop Shox2 Mice Show an Overexpression of Shox2 in the Temporomandibular Joint (TMJ)

**Figure 1:**
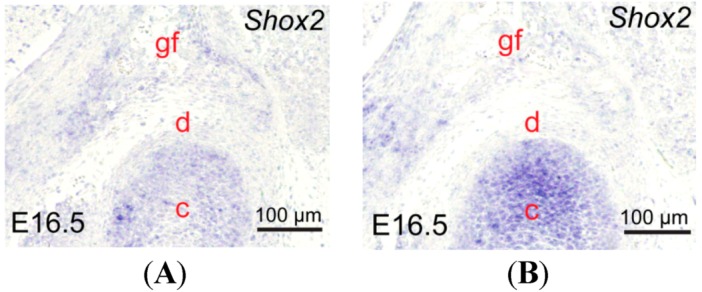
Expression of *Shox2* in the developing temporomandibular joint (TMJ) of *Wnt1-Cre*; *pMes-stop Shox2* mice. (**A**,**B**) *In situ* hybridization shows *Shox2* expression in the developing TMJ of E16.5 wild type (**A**) and *Wnt1-Cre*; *pMes-stop Shox2* embryo (**B**). c, condyle; d, disc; and gf, glenoid fossa.

*Shox2*, expressed in the developing temporomandibular joint (TMJ), plays an important role in the process of TMJ development. We aimed to determine if the expression of *Shox2* significantly up-regulated in the developing TMJ of *Wnt1-Cre*; *pMes-stop Shox2* mice. Our *in situ* assays show an overexpression of *Shox2* in the condyle of *Wnt1-Cre*; *pMes-stop Shox2* TMJ at embryonic day 16.5 (E16.5), compared to the wild type mice (Figure 1A,B).

### 2.2. Wnt1-Cre; pMes-stop Shox2 Mice Exhibit an Abnormal TMJ

**Figure 2:**
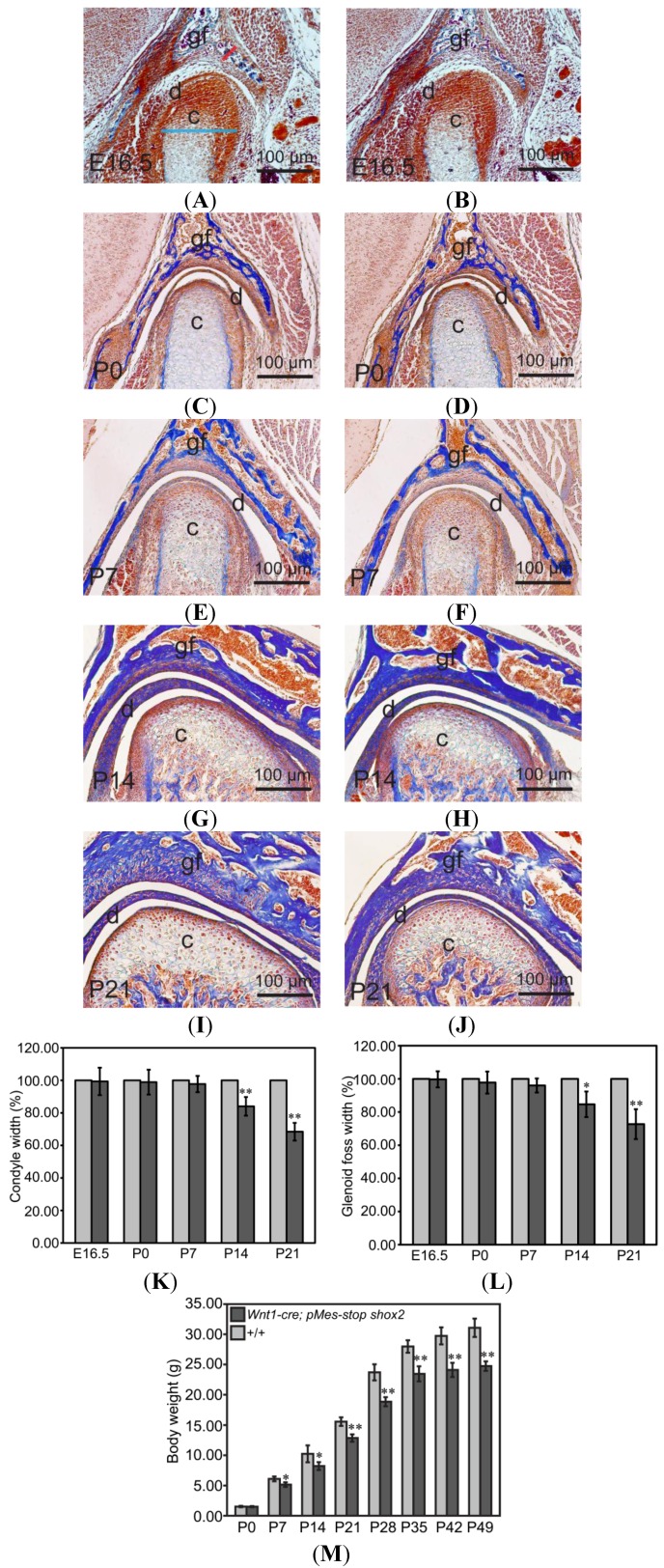
Histological analyses of TMJ of *Wnt1-Cre*; *pMes-stop Shox2* mice. (**A**–**E**) Coronal sections of the TMJ in wild type mice and *Wnt1-Cre*; *pMes-stop Shox2* mice at E16.5 (**A**,**B**), P0 (**C**,**D**), P7 (**E**,**F**), P14 (**G**,**H**), and P21 (**I**,**J**); (**K**,**L**) Comparison of condyle and glenoid fossa width of the TMJ in wild type and *Wnt1-Cre*; *pMes-stop Shox2* mice. Bright blue and red line segments in (**A**) point to the measured location of condyle and glenoid fossa on sections, respectively; and (**M**) Comparison of body weight at different time points in wild type and *Wnt1-Cre*; *pMes-stop Shox2* mice. Standard deviation is shown as error bars. * *p* < 0.05, ** *p* < 0.01. c, condyle; d, disc; and gf, glenoid fossa.

Histological analyses of TMJ showed no difference between wild-type mice and *Wnt1-Cre*; *pMes-stop Shox2* mice at E16.5, postnatal day 0 (P0) and P7 stage, and a phenotype of TMJ dysplasia from the P14 stage. To investigate the cellular and molecular alternations that may contribute to this phenotype of TMJ, we started with a time course analysis of changes in the width of the condyle and glenoid fossa in the TMJ. We focused on the greatest width part where the defect appears most significant. The average width of the wild type condyle and glenoid fossa at each time point (*n* = 3 for each time point) is defined as 100%. We found that at E16.5 (Figure 2A,B), P0 (Figure 2C,D) and P7 (Figure 2E,F), the width of the condyle and glenoid fossa appeared comparable between wild type and mutant mice (*n* = 3). However, at P14 (Figure 2G,H), the width of the *Wnt1-Cre*; *pMes-stop Shox2* condyle and glenoid fossa (*n* = 3) was reduced 15.97% ± 5.75% and 15.32% ± 7.69%, as compared to the wild type mice (Figure 1K,L). At P21 (Figure 2I,J), the *Wnt1-Cre*; *pMes-stop Shox2* condyle and glenoid fossa (*n* = 3) was further reduced 31.58% ± 5.45% and 27.30% ± 9.01% compared to the wild type mice (Figure 2K,L). These results indicate that the condyle and glenoid fossa dysplasia occurs primarily in the second week after birth.

To assess the effect of the condyle and glenoid fossa dysplasia on the body weight in *Wnt1-Cre*; *pMes-stop Shox2* mice, we measured the changes of body weight at different time points (P0, P7, P14, P21, P28, P35, P42 and P49) (Figure 2M). The results show a comparable body weight for both wild type and *Wnt1-Cre*; *pMes-stop Shox2* mice at birth, but this differs significantly at P7 and at other time points, indicating that the changes of condyle and glenoid fossa in *Wnt1-Cre*; *pMes-stop Shox2* mice may affect the function of TMJ, resulting in the loss of body weight, and develop a wasting syndrome, clinically defined as TMJ disorders.

### 2.3. Wnt1-Cre; pMes-stop Shox2 Mice Display Normal Gene Expression in the Developing TMJ

**Figure 3:**
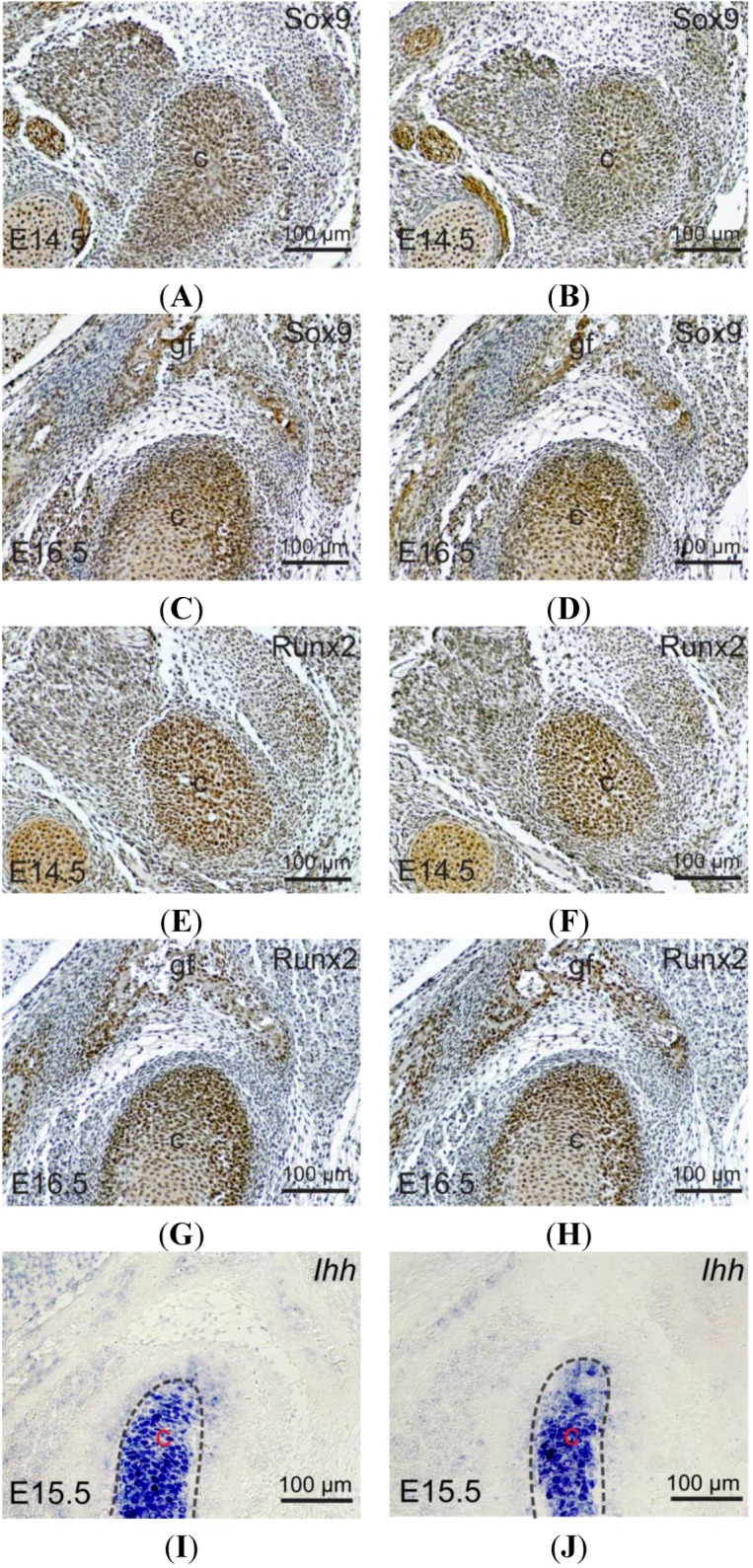
Expressions of Sox9, Runx2 and *Ihh* in the developing TMJ of *Wnt1-Cre*; *pMes-stop Shox2* mice. (**A**–**D**) Immunohistochemical staining shows Sox9 expression in the developing condyle of E14.5 (**A**,**B**) and E16.5 (**C**,**D**) wild type and *Wnt1-Cre*; *pMes-stop Shox2* embryo; (**E**–**H**) Immunohistochemical staining shows Runx2 expression in the developing TMJ of E14.5 (**E**,**F**) and E16.5 (**G**,**H**) wild type and *Wnt1-Cre*; *pMes-stop Shox2* embryo; and (**I**,**J**) *In situ* hybridization shows *Ihh* expression in the developing condyle of E15.5 wild type and *Wnt1-Cre*; *pMes-stop Shox2* embryo. c, condyle; and gf, glenoid fossa.

Previous reports show that tissue-specific inactivation of *Shox2* in the cranial neural crest cells leads to TMJ dysplasia and ankylosis, accompanied by significant down-regulation of *Sox9*, *Runx2* and *Ihh* [2]. We sought to determine if the expression of these genes were altered in the developing TMJ of *Wnt1-Cre*; *pMes-stop Shox2* mice. Our immunohistochemical assays display no alteration of Sox9 and Runx2 expression levels in the wild type and *Wnt1-Cre*; *pMes-stop Shox2* TMJ at E14.5 and E16.5 (Figure 3A–H). On the other hand, *in situ* hybridization shows that comparable *Ihh* expression is also retained in the *Wnt1-Cre*; *pMes-stop Shox2* TMJ at E15.5 (Figure 3I,J). These observations suggest that overexpression of *Shox2* does not affect early TMJ development.

### 2.4. Reduction of Extracellular Matrix (ECM) Proteins in the Wnt1-Cre; pMes-stop Shox2 TMJ

**Figure 4:**
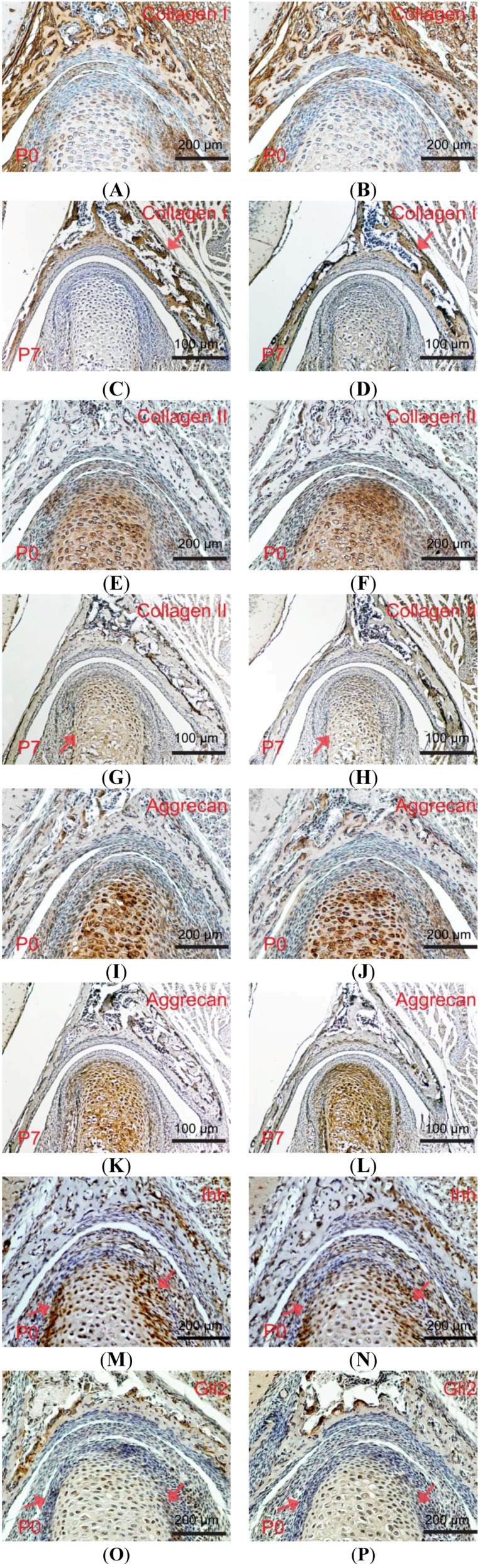
Expressions of extracellular matrix, Ihh and Gli2 in the postnatal TMJ of *Wnt1-Cre*; *pMes-stop Shox2* mice. (**A**–**L**) Expressions of Col I (**A**–**D**), Col II (**E**–**H**), and Aggrecan (**I**–**L**) in the TMJ of P0 and P7 wild type (**A**,**C**,**E**,**G**,**I**,**K**) and *Wnt1-Cre*; *pMes-stop Shox2* (**B**,**D**,**F**,**H**,**J**,**L**); (**M**–**P**) Immunohistochemical staining on coronal sections of P0 wild type (**M**,**O**) and *Wnt1-Cre*; *pMes-stop Shox2* (**N**,**P**) TMJ shows Ihh (**M**,**N**) and Gli2 (**O**,**P**) protein expression and distribution. Arrow heads point to the condyle and glenoid fossa where expression is altered.

The articular cartilage is composed of chondrocyte and extracellular matrix (ECM) components including collagens, proteoglycans (PGS) and glycosaminoglycans (GAGs) [15]. These ECM components play an important role in resistance to compressive forces and in maintaining the tensile properties of the tissue. Alterations of the ECM components are associated with cartilage degeneration [16], so we wondered if changes in the ECM components would account for the dysplasia of condyle and glenoid fossa in the *Wnt1-Cre*; *pMes-stop Shox2* TMJ. We examined the expressions of several ECM components including Collagen type I (Col I), Collagen type II (Col II), and Aggrecan using P0 and P7 mice (Figure 4A–L). Immunohistochemcal analyses show that the expressions of Col I, Col II, and Aggrecan are comparable between wild type and *Wnt1-Cre*; *pMes-stop Shox2* mice at P0 stage. However, at P7 stage, the expression of Col I is reduced in the glenoid fossa, and Col II expression is also decreased in the condyle in the TMJ of *Wnt1-Cre*; *pMes-stop Shox2* mice. In addition, Aggrecan expression is not affected in the condyle of *Wnt1-Cre*; *pMes-stop Shox2* TMJ as compared to the wild type mice. Thus, the reduction of Col I and Col II expressions could alter the ECM composition and contribute to the dysplasia of condyle and glenoid fossa in the *Wnt1-Cre*; *pMes-stop Shox2* TMJ.

### 2.5. Down-Regulated Ihh Signaling Pathway in the Wnt1-Cre; pMes-stop Shox2 TMJ

Ihh is critical for the completion of postnatal TMJ growth and organization. To determine if it affected the dysplasia of the condyle and glenoid fossa in *Wnt1-Cre*; *pMes-stop Shox2* mice, we investigated the protein levels of Ihh and Gli2 by immunohistochemistry at the P0 stage (Figure 4M–P). In the *Wnt1-Cre*; *pMes-stop Shox2* mice, Ihh and Gli2 was significantly down-regulated compared to the wild type mice. Thus, the down-regulation of Ihh and Gli2 expressions can act as a promoter to the formation of condyle and glenoid fossa dysplasia in *Wnt1-Cre*; *pMes-stop Shox2* mice.

### 2.6. Up-Regulated Matrix Metalloproteinases (MMPs) Activity in the Wnt1-Cre; pMes-stop Shox2 TMJ

**Figure 5:**
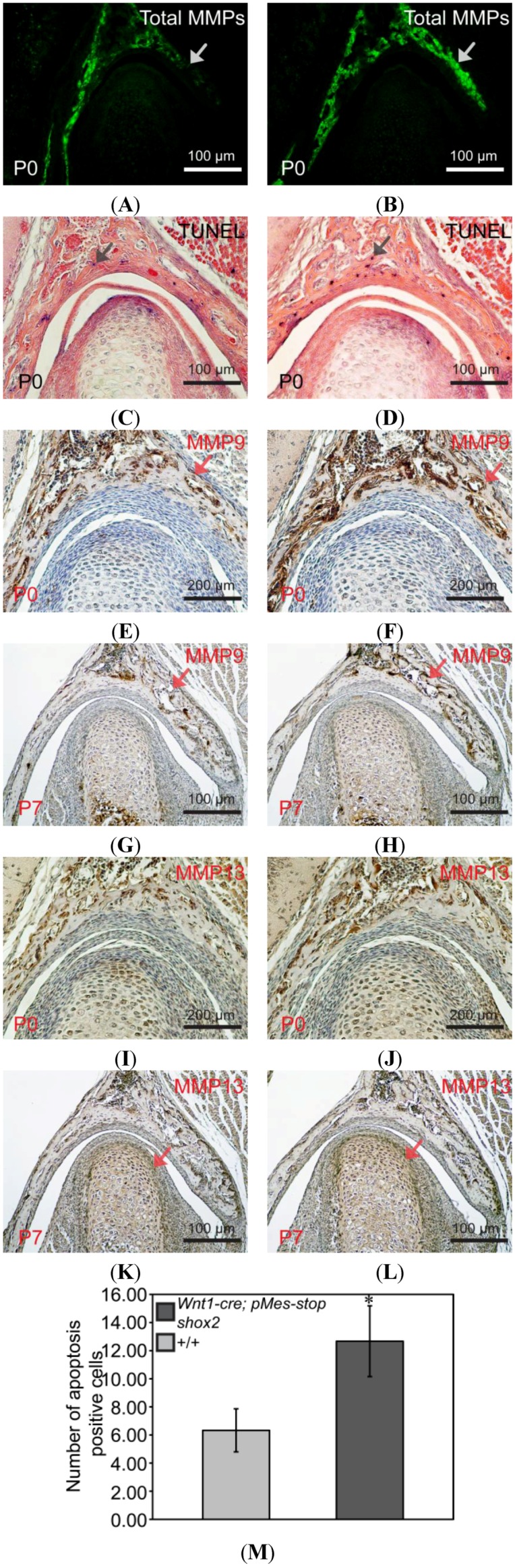
Altered expressions of MMPs and apoptosis in the postnatal TMJ of *Wnt1-Cre*; *pMes-stop Shox2* mice. (**A**,**B**) *In situ* zymography on coronal sections of wild type (**A**) and *Wnt1-Cre*; *pMes-stop Shox2* (**B**) TMJ shows MMPs activity. The ectopic MMPs activity in the glenoid fossa (arrow heads) of the *Wnt1-Cre*; *pMes-stop Shox2* TMJ; TUNEL assay on coronal sections of P0 wild type (**C**) and *Wnt1-Cre*; *pMes-stop Shox2* (**D**) TMJ shows an increase of apoptotic cells in the glenoid fossa of the *Wnt1-Cre*; *pMes-stop Shox2* TMJ. Arrow heads in (**C**,**D**) point to the apoptotic cells in the glenoid fossa; (**E**–**L**) Immunohistochemical staining on coronal sections of wild type (**E**,**G**,**I**,**K**) and *Wnt1-Cre*; *pMes-stop Shox2* (**F**,**H**,**J**,**L**) shows expressions of MMP9 (**E**–**H**) and MMP13 (**I**–**L**). Arrow heads in (**E**–**H**,**K**,**L**) point to the protein levels of MMP9 and MMP13in the condyle and glenoid fossa where expression is altered; (**M**) Comparison of numbers of apoptotic cells in the glenoid fossa between wild type and *Wnt1-Cre*; *pMes-stop Shox2* mice. Standard deviation is shown as error bars. * *p* < 0.05.

Matrix metalloproteinases (MMPs) are zinc-dependent endopeptidases capable of degrading ECM, including collagens and Aggrecan. We asked if the reduced levels of Col I and Col II in the *Wnt1-Cre*; *pMes-stop Shox2* TMJ were a consequence of enhanced MMPs activities. To test this possibility, we first conducted *in situ* zymography to determine total MMPs activity in P0 wild type and *Wnt1-Cre*; *pMes-stop Shox2* TMJ. As shown in Figure 5A,B, while strong MMPs activity is found in the glenoid fossa, and an above background level of MMPs activity is also seen in the condyle. Consistent with this enhanced total MMPs activity, we found that MMP9 and MMP13, which have been shown to be elevated in the TMJ osteoarthritis and are capable of degrading collagens and Aggrecan [17,18], exhibit enhanced expression in the *Wnt1-Cre*; *pMes-stop Shox2* TMJ at P0 and/or P7 stage (Figure 5E–L). These results indicate that enhanced MMPs activities are responsible for the reduction of ECM components, which may ultimately lead to the dysplasia of condyle and glenoid fossa in *Wnt1-Cre*; *pMes-stop Shox2* mice.

### 2.7. Enhanced Apoptosis in the Condyle of the Wnt1-Cre; pMes-stop Shox2 TMJ

**Figure 6:**
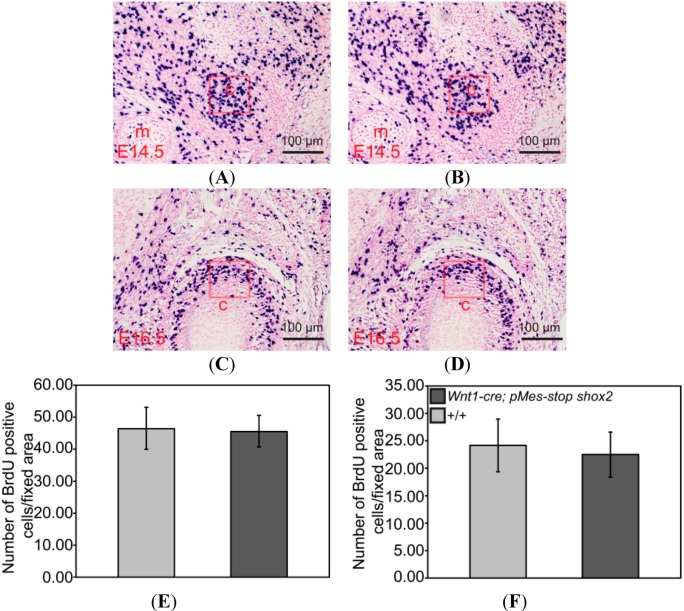
Changes of proliferation in the TMJ of *Wnt1-Cre*; *pMes-stop Shox2* mice. (**A**–**D**) BrdU labeling of cell proliferation in the condylar condensation of an E14.5 (**A**,**B**) and E16.5 (**C**,**D**) in wild type and *Wnt1-Cre*; *pMes-stop Shox2* embryo; (**E**,**F**) Comparison of numbers of BrdUpositive cells in the fixed area (red frame) of the condylar primoridain between wild type (**E**) and *Wnt1-Cre*; *pMes-stop Shox2* embryos (**F**). Standard deviation is shown as error bars.c, condyle; and m, Meckel’s cartilage.

We further examined if there existed cellular defects that could potentially contribute to the phenotype of condyle and glenoid fossa in the *Wnt1-Cre*; *pMes-stop Shox2* TMJ. Since apoptosis, or programmed cell death, is known to be responsible for tissue degeneration correlated with the severity of condyle and glenoid fossa pathologic processes [19,20], we determined if there was any alteration in cell apoptosis in the TMJ by TUNEL assay. Indeed, our results demonstrate a dramatically increased number of apoptotic cells in the glenoid fossa of *Wnt1-Cre*; *pMes-stop Shox2* TMJ compared to that in the wild type at P0 (*n* = 3) (Figure 5C,D,M), indicating a contribution of apoptosis to the glenoid fossa dysplasia in *Wnt1-Cre*; *pMes-stop Shox2* mice. In contrast, BrdU labeling assay revealed unaltered cell proliferation rate in the condyle (*n* = 3) of the *Wnt1-Cre*; *pMes-stop Shox2* TMJ compared to the wild type at E14.5 and E16.5 (Figure 6A–F).

## 3. Discussion

*Shox2* and *Shox*, found only in vertebrates, are implicated to play a role in the development of the internal skeleton and its related structures [21]. In humans, mutations of *Shox* gene function have been associated with a series of short-stature conditions, such as Léri-Weill dyschondrosteosis, Turner syndrome, and Langer dysplasia, which exhibit abnormalities in the skeletal development [22,23]. *Shox2* expression has also been found in the development of limbs in a complementary pattern to that of *Shox* [21]. However, any known syndrome that has been linked to *Shox2* mutations remains unclear thus far. In mice, targeted inactivation of *Shox2* leads to the virtual elimination of the stylopod in developing limbs [8,9]. A failure in growth, chondrogenesis, and endochondral ossification occurred in the mutant stylopodial cartilaginous element, accompanied by a down-regulation of several genes that are known to be essential for skeletogenesis, including *Runx2*, *Runx3* and *Ihh*. Indeed, besides the limb phenotype, *Shox2*-deficient mice exhibit severe defects in a number of developing organs (heart and palate), as well as the TMJ that exhibits dysplasia and ankylosis [10,11,12]. These observations demonstrate a crucial role for the *Shox2* genes in the development of the long bone that undergoes the endochondral ossification. Our previous studies support that inactivation of *Shox2* leads to the abnormal development of TMJ [2,14], and the overexpression of *Shox2* in the heart results in abnormal cardiac formation [24]. Therefore, we assume that the overexpression of *Shox2* may affect the TMJ development.

The changes of the ECM composition are associated with pathological processes of cartilage degeneration in TMJ osteoarthritis, accompanied by the up-regulation of MMPs [17,25,26]. Cartilage degeneration is a characteristic feature that results from the loss of collagens and PGS. It is well established that elevated MMPs activities are responsible for degradation of the ECM in the processes of TMJ osteoarthritis. Our results show that the phenotype of condyle and glenoid fossa dysplasia, a congenital non-pathological degenerative defect, is associated with reduced amounts of Col I, Col II, Ihh and Gli2 in the *Wnt1-Cre*; *pMes-stop Shox2* TMJ and an increase of apoptosis activity. The fact that enhanced MMPs activity was observed in the condyle and glenoid fossa of the *Wnt1-Cre*; *pMes-stop Shox2* TMJ suggests a similar mechanism for this non-pathological degenerative process. Although it is currently unclear whether the reduction in Ihh and Gli2 expression is a causative factor in the elevated MMPs activity in postnatal *Wnt1-Cre*; *pMes-stop Shox2* TMJ, it is consistent with an important role for Ihh signaling pathway in maintaining the proper structure and tissue homeostasis of postnatal TMJ [9], and warrants future investigation.

Congenital TMJ ankylosis has been reported in a patient with a short stature phenotype [27], however genetic alterations leading to congenital dysplasia and ankylosis of the TMJ are still unclear. Our results demonstrate a crucial role for *Shox2* in TMJ function and development, and provide evidence for a genetic association with congenital non-pathological cartilage degeneration. Histological analysis and an investigation of the molecular mechanisms involved in these processes will contribute not only to basic science but to clinical research.

## 4. Experimental Section

### 4.1. Mice Embryonic and Postnatal Heads Collection

All animal procedures were performed according to guidelines approved by the animal care committee of Fujian University of Traditional Chinese Medicine, Fuzhou, China. The *Wnt1-Cre*; *pMes-stop Shox2* mice from the cross of *Wnt1-Cre* mice [2] with *pMes-stop Shox2* mice [24] were provided from the lab of Yiping Chen. The presence of a vaginal plug indicates E0.5. Embryonic heads were fixed in 4% paraformaldehyde (PFA)/phosphate buffer saline (PBS) at 4 °C overnight. The heads from P0, P7, P14, and P21 mice were fixed and decalcified in Surgipath Decalcifier I (Leica Biosystems Richmond Inc., Richmond, CA, USA) for various days according to the age of the mouse.

### 4.2. Histological Analyses

Paraffin-embedded heads were sectioned at 10 µm with a microtome. To analyses the histology of TMJ, the serial sections were stained with Alizarin red/Alcian blue (Sigma, Saint Louis, MO, USA) staining according to standard procedures.

### 4.3. Immunohistochemistry and in Situ Hybridization

Paraffin-embedded heads were sectioned at 8 μm for immunohistochemistry and 10 μm for *in situ* hybridization. Immunohistochemical staining was performed using polyclonal antibody against Sox9 (1:500, ab26414), Runx2 (1:1000, ab76956), Aggrecan (1:500, ab36861), Col I (1:500, ab34710), Col II (1:200, ab53047), MMP9 (1:300, ab38898), MMP13 (1:50, ab75606), Ihh (1:200, ab39634), Gli2 (1:100, ab7195) from Abcam (Cambridge, MA, USA) according to the manufacturer’s instruction. *In situ* hybridization was performed on 10 μm paraffin sections with *Ihh* and *Shox2* digoxigenin-labeled probes as described [9,10].

### 4.4. In Situ Zymography

P0 mice heads were fixed in zinc-based fixative (ZBF) and embedded in optimum cutting temperature (OCT) compound. 10 μm Sections were incubated with dye-quenched (DQ)-gelatin (E12055, Molecular Probes Europe BV, Leiden, The Netherlands) according to the manufacturer’s instruction [28]. The gelatinolytic activity was observed as green fluorescence under a fluorescence microscopy (Fluorescence Microscope Model Axioskop 50, CarlZeiss, Oberkochen, Germany).

### 4.5. TUNEL Assay and Cell Proliferation Assay

Apoptosis of the TMJ was determined by *in situ* Cell Death Detection Kit and AP (alkaline phosphatase) from Roche (Mannheim, Germany). Paraffin-embedded heads were sectioned at 5 μm, and subjected to immunodetection for measurement of apoptosis according to the manufacturer’s protocol. Pregnant mice at E14.5 and E16.5 were injected with 1.5 mL labeling reagent/100 g body weight using the BrdU labeling and Detection Kit II from Roche (Mannheim, Germany) for 2 h. The Embryonic heads were fixed in Carnoy’s fixative, ethanol-dehydrated, paraffin-embedded, and sectioned at 5 μm. The sections were subjected to immunodetection for analysis of proliferation according to the manufacturer. Three independent experiments were conducted for TUNEL or cell proliferation assay.

### 4.6. Statistical Analysis

All experiments were repeated at least three times. Data were presented as mean ± Standard Deviation. Statistical analyses of the data were calculated using the Student’s *t*-test. *p*-values less than 0.05 were considered as statistically significant.

## 5. Conclusions

In summary, overexpression *Shox2* leads to the loss of ECM and the increase of cell apoptosis in the TMJ dysplasia by up-regulation of MMPs and down-regulation of the Ihh signaling pathway. Our results suggest that *Shox2* is essential for the process of TMJ development in mice, indicating that it may be further developed as a therapeutic target for the treatment of congenital TMJ dysplasia.

## Supplementary Files

Supplementary File 1
