# Supplementary material for: Overexpression of Shox2 Leads to Congenital Dysplasia of the Temporomandibular Joint in Mice
Source: Int J Mol Sci. 2014 Jul 24;15(8):13135–50. doi: 10.3390/ijms150813135 (PMC4159784; doi:10.3390/ijms150813135)
Supplement: Supplementary File 1 [file ijms-15-13135-s001.pdf]

Supplementary Information

1. Pires2-Ha-Shox2a Transfect in the NIH-3T3 Cells

NIH-3T3 murine fibroblast cells were cultured in Dulbecco's modified Eagle medium (DMEM) containing 10% fetal calf serum, 2 mM glutamine, 100 U/mL penicillin, and 100 mg/mL streptomycin at 37 °C and 5% CO_2 atmosphere. NIH-3T3 cells were seeded in 6-well cell culture plates with 2×10^5 cells per well, and were grown to semi-confluence. 20 µg of *Pires2-Ha-Shox2a* complexes with 20 µL of Lipofectin®, a cationic lipid transfection reagent, was used as a positive control and untreated cells were used as a negative control. After 72 h post-transfection, the expression of GFP in the cells was observed by a fluorescence microscope. Total RNA was isolated from post-transfection cells using TRIzol reagent (Invitrogen, Carlsbad, CA, USA). Reverse transcription was performed using random primers and SuperscriptTM III (Invitrogen, Carlsbad, CA, USA). Differential gene expressions were analyzed using the Agilent Technologies Oligonucleotide Microarrays.

2. Overexpression Shox2 Enhances the Matrix Metalloproteinases (MMPs) in the NIH-3T3 Cells

Qualitative analysis of transfection of NIH-3T3 cells by *Pires2-Ha-Shox2a* was carried out by fluorescence microscopy. Transfection efficiency was almost 30% at the end of 72 h. To explore the differential mRNA expression of overexpression *Shox2* on the NIH-3T3 cells using the Agilent Technologies Oligonucleotide Microarrays, we found that 199 genes are expressed in both transfection and untransfection cells, of these genes, 70 genes were down-regulated, while 129 genes were up-regulated in post-transfection cells compared to control cells at the fold-change ≥1.50. To focus on the effect of overexpression *Shox2* on the catabolism and anabolism of bone and cartilage, the alteration of *MMPs* was taken. The mRNA expressions of *MMP3* (1.79-fold), *MMP10* (1.58-fold) and *MMP13* (2.62-fold) associated with extracellular matrix (ECM) degeneration increased respectively in the post-transfection cells compared to control cells, indicating that MMPs alterations are responsible for the TMJ dysplasia in the *Wnt1-Cre*; *pMes-stop Shox2* mice.
